# T165. ULTRA-HIGH FIELD MORPHOMETRY IN DRUG-NAïVE FIRST EPISODE PSYCHOSIS

**DOI:** 10.1093/schbul/sby016.441

**Published:** 2018-04-01

**Authors:** Tushar Das, Kara Dempster, Michael Mackinley, Peter Jeon, Joe Gati, Jean Theberge, Ali Khan, Lena Palaniyappan

**Affiliations:** 1 University of Western Ontario; 2 London Health Sciences Center; 3 Robarts Research Institute

## Abstract

**Background:**

Structural neuroimaging studies report distributed grey matter volume (GMV) deficits in drug-naïve first episode psychosis (FEP), though their relevance to symptom burden and cognitive deficits is currently unclear. When compared to studies in medicated patients and/or patients with established later-stage of psychosis, the GMV deficits reported have been limited in both spatial distribution and effect size, indicating the possibility of stage-specific progression during the clinical course of psychosis. TOPSY (Tracking Outcomes of Psychosis) is one of the first studies intending to track the neurobiological trajectory using ultra-high field (7T) imaging starting from a drug-naïve first episode state. Here, we report the initial findings from the voxel-based morphometry (VBM) of GMV. To our knowledge, this is the first VBM report from drug-naïve FEP subjects obtained using a 7T MRI acquisition.

**Methods:**

We used ultra-high field (7 Tesla) MRI in 28 patients with FEP (satisfying criterion A of DSM-5 schizophrenia) and 18 controls, to evaluate differences in the grey matter. Volume in a voxelwise manner. FEP and controls were matched for age, sex and parental socioeconomic status. Patients were recruited at an early intervention unit (PEPP, London Ontario) and had active psychotic symptoms at the time of scanning. We also obtained abbreviated PANSS (8 items) scores to index the severity of psychosis. Analysis was done using SPM12, after DARTEL based registration and segmentation but without spatial smoothing. 2-tailed voxelwise T-test with FDR correction (p=0.05, 5% rate for false positives) was used. We used multiple regression analysis to predict the scores from processing speed measure (modified Symbol Substitution Test) and the severity of Delusions and Unusual Thought Content (P1 and G9), the 2 symptoms for which most subjects sought treatment in the first place.

**Results:**

Patients had a significant reduction in GMV in left fusiform gyrus (Hedge’s g = 1.98, T= 6.7), and increased GMV in the right precuneus (Hedge’s g = 1.63, T= 5.5) and lingual cortex (Hedge’s g = 1.19, T= 4.0). We did not find any other areas of significant GMV change. Of these 3 circumscribed GMV changes, reduced fusiform GMV was found among FEP patients with lower processing speed (ß=0.45, p=0.04), higher severity of delusions (ß=-0.43, p=0.049) and unusual thought content (ß=-0.59, p=0.01). Increased precuneus GMV was found among FEP patients with higher severity of delusions (ß=0.62, p=0.008) and unusual thought content (ß=0.50, p=0.03). Right lingual changes were not related to the severity of delusions or processing speed scores.

**Discussion:**

Our findings suggest that (1) GMV deficits are minimal in drug-naïve FEP subjects, with large effect-size changes concentrated around face processing (fusiform) region (2) GMV increases co-occur with GMV reduction especially in those with most severe delusions and cognitive deficits indicating a role for compensatory plasticity. Subtle early brain structural changes appear to predict symptom burden and cognitive deficits at the time of first clinical presentation with psychosis. Focusing on treatments that manipulate the structure of fusiform cortex could potentially reduce the severity of some of the early symptoms in FEP.

